# Cancer of the Uterus

**Published:** 1902-12

**Authors:** 


**Cancer of the Uterus.** By THOMAS STEPHEN CULLEN, M.B. Pp. xvi., 693. New York: D. Appleton and Company. 1900.

This volume is a welcome addition to the literature of gynæcological pathology. The plates and illustrations are most clear and very thoroughly described, and being in a large number of cases the result of photo-micrography, are free from the diagrammatic defects which it is difficult to avoid in drawings. The author gives a short resumé of the ways in which his material was obtained, and an interesting account of the technique of his methods of preparation. According to his description, his *rapid* method allows of the identification of a specimen fifteen minutes after its excision; the advantage to the patient gained by such a rapid confirmation of a diagnosis is obvious, even if the microscopical evidence only adds one more point to the completion of a diagnosis probably already more than provisional. We think the author is perhaps unduly optimistic as to the powers of the microscope as a diagnostic agent in these cases: we cannot imagine in practice an accidental or casual positive microscopical diagnosis being allowed to weigh against clinical signs. We have had personal experience of cases in which such a diagnosis has proved faulty in the light of subsequent clinical history; in two cases at least in our knowledge, patients in whom apparently conclusive evidence of the existence of malignant disease was given by microscopical sections, have survived without further developments for six and nine years respectively.

Unless the author's methods are far more complete than those in vogue with us, we should hesitate before accepting microscopical evidence, especially negative evidence derived from uterine scrapings, as conclusive unless backed by negative clinical evidence.

In addition to the pathological descriptions, operative procedures are very fully described, and interesting tables are given; chapters which will well repay perusal are those on symptomatology, prognosis, and etiology. As regards the last, the author, on the whole, is inclined to discount heredity: he considers traumatism, as evidenced by the frequent occurrence of squamous carcinoma of the cervix in parous women, an important factor, and does not consider that emdometritis is a distinct cause. Traumatism, in his opinion, does not play any large part in the causation of carcinoma of the corpus uteri.

We can only repeat that this work will be of the greatest assistance to every student of uterine pathology.

